# Inverted Takotsubo-Like Left Ventricular Dysfunction with Pulmonary Oedema Developed after Caesarean Delivery Complicated by Massive Haemorrhage in a Severe Preeclamptic Parturient with a Prolonged Painful Labour

**DOI:** 10.1155/2011/164720

**Published:** 2011-12-26

**Authors:** Hyejin Jeong, Seongheon Lee, Cheolwon Jeong, Jongun Lee, Seongtae Jeong, Sungsu Chung, Kyungyeon Yoo

**Affiliations:** ^1^Department of Anaesthesiology and Pain Medicine, Chonnam National University Medical School, 671 Jebongno, Donggu, Gwangju 501-757, Republic of Korea; ^2^Department of Physiology, Chonnam National University Medical School, Donggu, Gwangiu 501-190, Republic of Korea; ^3^Department of Anaesthesiology, School of Dentistry Chonnam National University, 671 Jebongno, Donggu, Gwangju 501-190, Republic of Korea

## Abstract

Inverted takotsubo cardiomyopathy (TTC), a variant of stress-induced cardiomyopathy, features transient myocardial dysfunction characterized by a hyperdynamic left ventricular apex and akinesia of the base. Herein, we describe a 38-year-old primigravida with severe preeclampsia who had active labour for 4 h followed by an emergency caesarean delivery. She developed postpartum haemorrhage due to uterine atony complicated by pulmonary oedema, which was managed with large-volume infusion and hysterectomy. Her haemodynamic instability was associated with cardiac biomarkers indicative of diffuse myocardial injury and echocardiographic findings of an “inverted” TTC. The patient was almost fully recovered one month later. Our case shows that a reversible inverted TTC may result from a prolonged painful labour. TTC should be listed in the differential diagnosis of the patient presenting with pulmonary oedema of unknown origin, especially in patients with severe preeclampsia.

## 1. Introduction

Stress-induced cardiomyopathy, also called takotsubo cardiomyopathy (TTC), is a transient stunned myocardium characterized by suspicion of an acute myocardial infarction in patients who have no angiographic stenosis upon coronary angiography [1, 2]. It has been in part attributed to severe physical and emotional stress leading to catecholamine surge [2], typically lasting for a few days to a few weeks with a good prognosis [3].

Labour pain may cause stress and emotional responses in a parturient [4]. In addition, preeclampsia has been associated with sympathetic overactivity [5]. Furthermore, a severe preeclampsia if any may precipitate a pulmonary oedema linked to fluid administration [6]. Herein, we present a case of TTC which occurred after a prolonged painful labour followed by an emergent caesarean delivery. There developed a postpartum haemorrhage secondary to uterine atony during the surgery, which was managed with large-volume infusion and hysterectomy.

## 2. Case Presentation

A 38-year-old primigravida (height, 155 cm; weight, 76 kg) presented for elective induction of labour at 40^+3^ weeks of gestation with oedema of both legs lasting the last 2 weeks. She had no significant past medical history except for chronic hepatitis B. No coronary risk factors were reported. On admission, her blood pressure was 150/90 mmHg and urinary protein level was as high as 500 mg/dL. Her blood tests revealed haemoglobin 11.9 g/dL, platelets count 106 × 10^9^/L, prothrombin time 11.0 s (normal range 9.8–13 s), international normalized ratio of 1.11, and activated partial thromboplastin time 47.7 s (normal range 26.5–41 s). Severe preeclampsia was diagnosed and MgSO_4_ 4 g i.v. plus 10 g intramuscularly was given initially as a loading dose, followed by 1 g/h as infusion for seizure prophylaxis.

After 6 h in hospital, labour was induced with insertion of extraovular catheter through the cervical canal of the uterus and spontaneous uterine contraction began 5 hours thereafter. When the cervix was 2.5 cm dilated, an infusion of oxytocin was started to augment labour and the membranes were ruptured 3 hours thereafter. Throughout the labour course including 4 h of active, her arterial pressure was stable in the range of 140–160 mmHg systolic, 90–100 mmHg diastolic. She declined epidural labour analgesia because of possible aggravation of the backache she had had.

Twenty-one hours after labour onset, when the cervix was 10 cm dilated, the foetal vertex did not descend past 1 station (arrest disorder) and the foetus developed deceleration (90 beats/min). The patient became dyspneic and diaphoretic and complained of sudden onset of severe left-sided chest pain. Her blood pressure was 160/90 mmHg. On electrocardiogram (ECG), a sinus tachycardia (100 beats/min) was noted with no ST-segment changes. She had been tolerating oral fluids but had taken nothing by mouth for more than seven hours. The decision was made to perform category-1 caesarean delivery. The potential risks and benefits of general, spinal, or epidural anaesthesia were explained to the patient, who accepted the anaesthetist's inclination towards general anaesthesia.

The patient received 30 mL of 0.3 M sodium citrate before induction of anaesthesia. Upon arrival in the operating theatre, the patient was positioned supine with left lateral tilt and routine monitoring devices including continuous EKG, noninvasive blood pressure, temperature, and pulse oximetry were applied. A 20-gauge catheter was placed into a radial artery and connected to a pressure transducer to measure blood pressure. Initial recordings showed a heart rate of 103 beats/min, blood pressure 170/110 mmHg, respiratory rate 25 breaths/min, and peripheral oxygen saturation (SpO_2_) 96% on air. After preoxygenation, a rapid sequence technique with remifentanil 1 *μ*g/kg, thiopentone 300 mg, and suxamethonium 75 mg was employed and cricoid pressure was applied. After endotracheal intubation, anaesthesia was maintained with 1.2% sevoflurane (end-tidal) and 50% nitrous oxide in oxygen, with vecuronium 6 mg for neuromuscular block. A female infant weighing 4000 g was born through a low transverse uterine incision with Apgar scores of 5 and 7 at one and five minutes, respectively, and the baby was transferred to the neonatal ICU for observation. After cord clamping, an oxytocin infusion of 40 units in 500 mL of normal saline was commenced at 125 mL/h. In addition, i.v. 0.15 mg/kg morphine, cefazolin 1 g, and midazolam 3 mg were given while nitrous oxide was increased to 70% and end-tidal sevoflurane concentration was reduced to 0.8%. Approximately 5 min after delivery, the uterus was found to be atonic and bleeding diffusely. The oxytocin infusion rate was increased to 240 mL/h after an i.v. bolus of carbetocin 100 *μ*g with continuous bimanual compression of the uterus. The fluid infusion rates were increased by pressurizing the infusion bags. Additional peripheral venous and central (left subclavian vein) venous assess was established with initial central venous pressure (CVP) value of 5.5 cm H_2_O. As the bleeding continued, bimanual uterine compression was combined with i.v. ergometrine 250 *μ*g and blood transfusion. Nevertheless, the uterus was still atonic with blood spurting from the lax uterus. There were no vaginal or cervical lacerations. The patient lost more than 2500 mL within 30 min and rapidly became haemodynamically unstable. Additional oxytocics including intramyometrial carboprost (1 mg in divided doses), and repeated doses of i.v. oxytocin 5 units with warmed fluids and blood products were given. However, this was inadequate to address the clinical situation and finally, decision was made to proceed to hysterectomy.

During the surgery, the systolic blood pressure was decreased to 70–90 mmHg and the pulse was tachycardic at 122–130 beats/min, despite the massive intravenous fluids and blood products combined with inotropic support (dopamine at 2 to 5 *μ*g/kg/min). Her SpO_2_ remained between 92% and 98% at FiO_2_ 0.5 during the operation. On physical examination, rales were detected in both lungs and diuresis was started with furosemide. Her haematocrit level dropped below 15%. A total of 6700 mL crystalloid, 2,000 mL colloid, 9 units packed red blood cells, 3 units fresh frozen plasma, and 8 units cryoprecipitate were administered during the 4.5 h surgical procedure. Intraoperative blood loss was estimated to be 5,800 mL and urine output was about 260 mL in the operating theatre. Arterial blood gas analysis on an FiO_2_ of 0.5 revealed a pH of 7.25, PaO_2_ 11.89 kPa (89 mmHg), PaCO_2_ 5.60 kPa (42 mmHg), HCO_3_ 18.4 mmol/L, and base excess −8.8 mmol/L at the end of operation (an acute onset of hypoxemia with a ratio of arterial PaO_2_ to FiO_2_ of <200). Tentative diagnosis of pulmonary oedema related to fluid overload was made.

**Figure 1:**
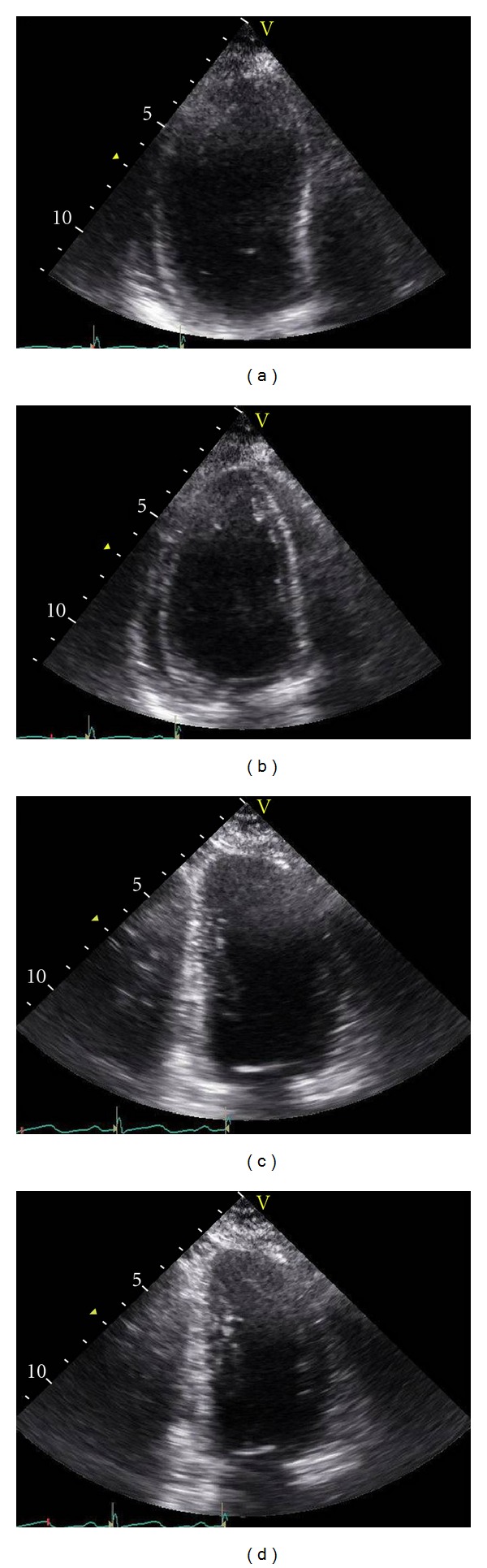
End-diastolic (a) and end-systolic (b) frames of two chamber view on initial echocardiography taken 12th day showing severe left ventricular systolic dysfunction with akinesia of the left ventricular base and mid-portion, and hypercontractility of the apex, and a followup echocardiography at the end of diastole (c) and systole (d) taken 28th days showing nearly normalized cardiac function without regional wall motion abnormalities.

 On completion of the procedure, the patient was transferred intubated and sedated to the intensive care unit (ICU) for further management. Initial recordings on arrival in the ICU were heart rate 132 beats/min, blood pressure 125/76 mmHg, and CVP 18.5 cm H_2_O with dopamine infusion at 5 *μ*g/kg/min. Because of the tachycardia dopamine was switched to dobutamine (3–5 *μ*g/kg/min) which was continued until the weaning of inotropic support. The patient was mechanically ventilated with a tidal volume of 8 mL/kg at an assisted control rate of 12 breaths/min and a 10 cm H_2_O positive end-expiratory pressure with supportive care for pulmonary oedema, including oxygen, furosemide (20 mg q 4 hour), and fluid restriction. A chest X-ray taken immediately after ICU admission showed extensive bilateral pulmonary infiltrates in both hilar areas and pleural effusion in both lungs with normal lung volume and normal heart size. On the postoperative day 2, the patient became haemodynamically stable and was weaned off inotropic support. On the day of admission, urine output was about 80 mL/h but her renal function continued to deteriorate, and oliguria (creatinine 369.6 *μ*mol/L) set in by day 4. Continuous renal replacement therapy (CRRT) was initiated to treat the acute pulmonary oedema as well as acute renal failure. Over the next 3 days, renal function gradually improved, and CRRT was discontinued on the third day, and the patient was weaned off the ventilator on the next day. However, she was still tachypneic (22–30 breaths/min) with peripheral oxygen saturation of 94–98% on 5 L/min of oxygen via nasal prongs over the following 4 days. Therefore, a cardiac workup was initiated, and an initial transthoracic echocardiogram (TTE) on the 12th day revealed severe left ventricular (LV) systolic dysfunction and ejection fraction of 23.5% with extensive akinesia of the LV base extending to the midcavity of the heart, together with preserved apical function (Figures 1(a) and 1(b)). ECG on the same day revealed nonspecific ST-T wave changes with normal sinus rhythm, and peak troponin I was slightly raised to 0.88 ng/mL (reference range, 0–0.1 ng/mL) with normal creatine kinase-MB fraction level 2.2 U/L (reference range, 2.3–9.5 U/L) and proBNP level >35000 pg/mL. Based on the patient's history of chest discomfort when she was in active labour along with absence of cardiovascular risk factors, mild cardiac enzyme elevation, and the characteristic TTE findings, she was diagnosed with inverted TTC. She was started on heparin, a ß-adrenergic blocker, and an angiotensin-converting enzyme inhibitor, after which her clinical condition improved steadily. On the 14th day, the followup TTE showed slight improvement in LV function with an ejection fraction of 29.4%. On the 28th day, TTE showed a return to near normal LV function (ejection fraction 54.6%) with resolution of the basal wall motion abnormalities (Figures 1(c) and 1(d)). The following hospital course was uneventful. She was discharged home 38 days after surgery. The patient remained asymptomatic at the 5-month followup with complete recovery of LV systolic function on TTE (ejection fraction 65.0%).

## 3. Discussion

We present a case of unrecognized inverted TTC resulting in pulmonary oedema in a severe pre-eclamptic woman who had caesarean delivery following a prolonged painful labour complicated by massive postpartum haemorrhage secondary to uterine atony. She experienced a severe sudden onset of substernal chest pain during the active labour accompanied by mild dyspnea and palpitations. The correct diagnosis was delayed by the initial belief that the symptoms were due to fluid overload. The patient was then treated with diuretics, mechanical ventilation, and fluid restriction. Our misdiagnosis was in fact encouraged by high risk of pulmonary oedema in patients with severe preeclampsia [6] and massive fluid loading during the surgery.

 In the perioperative period, pulmonary oedema has been observed after a variety of inciting events, including anaphylaxis [7], severe central nervous system trauma (neurogenic pulmonary oedema) [8], acute lung injury [9], upper airway obstruction (negative pressure pulmonary oedema) [10], fluid overload, and acute cardiac events. In our case, neither the evidence of neurogenic pathology nor the signs or symptoms of anaphylaxis or airway obstruction were noted. Nevertheless, since it is thought that a catecholamine surge leads to systemic and pulmonary vasoconstriction, increase in pulmonary hydrostatic pressure, and increase in permeability of pulmonary capillaries in neurogenic pulmonary edema [8], we cannot rule out the contribution of catecholamine released exaggeratively in response to labour stress in the development of pulmonary edema in our case of severe preeclampsia [5]. A transfusion-related acute lung injury was also unlikely, as it should have been resolved within 48 to 96 hours of the transfusion [8]. Now that the time course of clinical and radiologic recovery was not ultimately consistent with the idea of fluid overload, we performed a cardiac workup in which initial TTE revealed findings compatible with inverted TTC. In view of the patient's history of chest discomfort in the absence of cardiovascular risk factors, mild cardiac enzyme elevation, and TTE findings, she was diagnosed with TTC.

A severe preeclampsia alone can cause pulmonary oedema due to increased capillary permeability, increased sympathetic tone, decreased colloid oncotic pressure, and an elevated afterload [6]. Therefore, management of fluid balance before, during, and after delivery is a challenge for the clinician in patients with severe preeclampsia. Our patient received a huge load of fluids and blood products during the surgery. Furthermore, a TTC was complicated, which may have led to congestion in the pulmonary circulation. It is likely that massive volume loading and LV dysfunction secondary to TTC may have led to the development of pulmonary oedema in our case with an already elevated afterload due to preeclampsia and a physiologically increased intravascular volume and cardiac output owing to pregnancy. It should have been required to perform echocardiography to guide fluid management and specifically to exclude peripartum cardiomyopathy or TTC shortly after admission to the ICU. Indeed, echocardiography is highly recommended for evaluation of all pregnant women with pulmonary oedema because it accurately differentiates patients with respect to the presence and the type of cardiac dysfunction [11]. Unfortunately, the echocardiography was not available in ICU of our institution by then, which resulted in delayed diagnosis and treatment of TTC.

Monitoring of CVP and pulmonary capillary artery occlusion pressure (PAOP) has been also advised in the pre-eclamptic patient for aggressive fluid resuscitation and pulmonary oedema. However, the use of CVP to estimate PAOP is limited by poor correlation between the values in women with preeclampsia [12]. It has been suggested that the patient with severe complicated preeclampsia should be managed either clinically or with a pulmonary artery catheter [13]. Nevertheless, until recently, randomized trials that demonstrate a clear benefit of this catheter-directed care are lacking, and much of the available data are conflicting.

The mechanisms underlying the transient severe myocardial dysfunction (TTC) should be multifactorial. Among others, a catecholamine-mediated myocardial stunning has been highly favoured, since myocardial function returns to normal within a few days to a few weeks [14, 15]. Indeed, an extreme physiologic or emotional stress leading to catecholamine surge has been reported to precipitate into TTC [2]. The patient had experienced 4 h of active labour without analgesia in a course of 21 h of labour in our case. Apart from the prolonged painful labour, she should have had anxiety and exhaustion. Furthermore, preeclampsia is characterized by increased sympathetic activity with elevated plasma norepinephrine levels [5]. In fact, she complained of sudden onset of severe chest discomfort and shortness of breath just prior to the operation. The prolonged active labour in the presence of severe preeclampsia may have led to the development of TTC before the start of the operation.

TTC is being increasingly recognized and even reported in relatively young women in the peripartum period [16, 17], although it is most often encountered in postmenopausal women, soon after a psychic and/or physical stress. Our case illustrates an important link between painful labour in a patient with preeclampsia and an acute cardiac event, suggesting labouring women lacking adequate analgesia may be at a risk of exaggerated stress response, thus precipitating a transient cardiomyopathy. Importantly, an attention appears to be paid to the pain control during active labour to minimise the stress and catecholamine release, and to consider TTC in listing the differential diagnosis of the patient presenting with cardiac dysfunction complicated by pulmonary oedema of unknown origin, especially in patients with preeclampsia.

Neuraxial anaesthesia is the standard for elective caesarean delivery in many countries and has become a preferred technique to provide anaesthesia for caesarean delivery even among women with severe preeclampsia [18]. However, in our case, general anaesthesia was chosen because a delay in establishing regional anaesthesia would not be ethically justifiable in an emergency surgery. Instead, we used remifentanil 1.0 *μ*g/kg to prevent a pressor response to tracheal intubation which is our routine in parturients with severe preeclampsia, since an abrupt increase in arterial pressure, although transient, can lead to cerebral oedema and haemorrhage, and cardiac failure with pulmonary oedema [19]. Remifentanil may be ideally suited for use in obstetrics because of an extremely short duration of its action [20]. Indeed, remifentanil has been shown to blunt cardiovascular responses to intubation with minimal neonatal respiratory depression in severe pre-eclamptics undergoing cesarean delivery [21].

The effectiveness of MgSO_4_ for seizure prophylaxis and control has been well admitted in severe preeclampsia [22]. However, its use in the presence of a large positive fluid balance may culminate in a development of pulmonary oedema in antenatal patients treated for preterm labour [22]. One may not rule out the role of magnesium in the development of pulmonary oedema in our case. In addition, MgSO_4_ in high doses may prolong the effect of nondepolarizing muscle relaxants and thus monitoring of neuromuscular function is essential in patients given large dose of MgSO_4_ [23].

In conclusion, a pulmonary oedema following caesarean delivery, particularly when associated with increased CVP, requires immediate investigation with TTE in patients with severe preeclampsia. TTC should be listed in the differential diagnosis.
